# Sources, Components, Structure, Catalytic Mechanism and Applications: a Critical Review on Nicotinate Dehydrogenase

**DOI:** 10.4014/jmb.2302.02011

**Published:** 2023-03-06

**Authors:** Zhi Chen, Xiangjing Xu, Xin Ju, Lishi Yan, Liangzhi Li, Lin Yang

**Affiliations:** 1 School of Chemistry and Life Science, Suzhou University of Science and Technology. Suzhou City 215009, Jiangsu Province, P.R. China; 2 College of Life Sciences, Jiangxi Normal University. Nanchang City 330022, Jiangxi Province, P.R. China

**Keywords:** Neonicotinoid insecticides (NIs), nicotinate dehydrogenase (NDHase), 6-hydroxynicotinic acid, 2-chloro-5-methylpyridine, microbial conversion of nicotinate

## Abstract

Plant-derived insecticide-neonicotinoid insecticides (NIs) played a crucial role in the development of agriculture and food industry in recent years. Nevertheless, synthesis of these nitrogen-containing heterocyclic compounds with an effective and greener routing remains challenging especially to the notion raise of “green chemistry” and “atom economy”. While bio-catalyzed methods mediated by nicotinate dehydrogenase (NDHase) then provide an alternative. The current review mainly focuses on the introduction of sources, components, structure, catalytic mechanism and applications of NDHase. Specifically, NDHase is known as nicotinic acid hydroxylase and the sources principally derived from phylum *Proteobacteria*. In addition, NDHase requires the participation of the electron respiratory chain system on the cell membrane. And the most important components of the electron respiratory chain are hydrogen carrier, which is mainly composed of iron-sulfur proteins (Fe-S), flavin dehydrogenase (FAD), molybdenum binding protein and cytochromes. Heterologous expression studies were hampered by the plasmid and host with high efficiency and currently only *Pseudomonas entomophila* L48 as well as *Comamonas testosterone* was successfully utilized for the expression of NDHase. Furthermore, it is speculated that the conjugate and inductive effects of the substituent group at position 3 of the substrate pyridine ring exerts a critical role in the hydroxylation reactions at position 6 concerning about the substrate molecular recognition mechanism. Finally, applications of NDHase are addressed in terms of pesticide industry and wastewater treatment. On conclusion, this critical review would not only deepen our understanding of the theory about NDHase, but also provides the guideline for future investigation of NDHase.

## Introduction

Pesticides, a pivotal input in agricultural production, have made great contributions to the development of agriculture and the supplies of human food. However, the extensive use of pesticides not only affects peoplés physical and mental health, but also causes serious damage to the ecological environment [1, 2]. For example in China, the utilization of pesticides far exceeds the production, and the amount of pesticides used per unit area is twice that of developed countries [3]. Among them, 70%-80% of the pesticides will directly penetrate the environment, causing pollution to soil, agricultural products, surface and groundwater, and further enter the human body through the enrichment of the biological chain [4]. As one kind of pesticides, insecticides can be divided into four major categories including organophosphates, pyrethroids, carbamates and organochlorines according to the chemical composition and source of chemical structure [5].

Nitrogen-containing heterocyclic compounds are widely present in industrial wastewaters such as coking, chemical, dyes and pesticides [6]. Common nitrogen-containing heterocyclic compounds mainly include pyrrole, pyridine, indole, quinoline, isoquinoline and their derivatives [7]. Compared with aliphatic or aromatic compounds, nitrogen-containing heterocyclic compounds are not easily metabolized in nature, and possess poor biodegradability. In addition, many of them harbor the effects of mutagenesis, carcinogenesis and teratogenesis, which are potentially harmful to human health and the ecological environment [8]. Furthermore, the production and utilization of highly toxic organophosphorus pesticides seriously affected the ecological balance. Therefore, the sale and utilization of organophosphorus insecticides has been completely prohibited since 2007 and was replaced by the development of high-efficiency as well as low-toxicity insecticides [9]. Insecticides with wide spectrum, high safety and high activity are herein sought by scientists and entrepreneurs, and the development of neonicotinoid insecticides has become a reality.

## Neonicotinoid Insecticides

Nicotine belongs to the plant-derived insecticide. In 1690, it was found that tobacco extract could kill *Stephanitis nashi*, and then in 1829, it was determined that the active ingredient was nicotine [10]. As a pure biological source pesticide, nicotine possesses a lot of advantages including strong affinity with nature, good selectivity, no pollution, unique insecticidal mechanism and high activity and acted as blocking the normal conduction of the central nervous system by controlling the nicotinic acetylcholinease receptors in the nervous system, resulting in paralysis and death [11]. In addition, nicotine also has the fumigation and poisoning effects with fast efficacy, volatility, short validity period as well as short duration of effect. It is one of the preferred pesticide varieties for the development and production of green products.

**Fig. 1:**
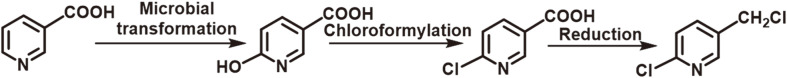
Microbial transformation plus simple chemical reactions for the synthesis of 2-chloro-5-chloromethylpyridine.

In the 1940s, nicotine was one of the mainstays of pesticides, but with the rapid development of chemical pesticides and the shrinking of nicotine due to the emergence of resources, activity, toxicity and other issues, nicotine can no longer meet requirements. Under the circumstance that the creation of novel pesticides is becoming more and more challenging, scientists successfully synthesized a series of novel pesticides by modifying the structure of natural source substances. Consequently, neonicotinoid insecticides represented by imidacloprid came into being. At present, the commercialized neonicotinoid insecticides mainly include imidacloprid, acetamiprid, thiamethoxam, thiacloprid, clothianidin, dinotefuran and so forth [12] (Fig. 1).

Imidacloprid was jointly developed by Bayer Co. Ltd. and Special Co. Ltd. (Japan) in early 1985 [13]. It has the characteristics of high efficiency, broad spectrum, low toxicity, quick initial effects, long lasting effects, high systemic absorption and no cross resistance, which can be widely used not only for seed treatment, but also for foliar spraying, stem and leaf treatment and soil treatment. Imidacloprid was mainly used to control aphids, thrips, whitefly and other pests. At present, it has been widely used in more than 60 kinds of crops in more than 100 countries, and is a new pesticide variety with fierce competition in the market.

Acetamiprid, developed by SODA Co. Ltd. (Japan) in 1996, is also a nicotinic insecticide [14]. However, due to the good effect and mature technology of the similar insecticide imidacloprid, its development is limited to a certain extent. At the same time, due to the widespread use of imidacloprid, the problem of pest resistance is shifting more and more serious. With the continuous improvement of acetamiprid technology, the pesticide will have broad practical prospects.

Several large pesticide companies all have invested in the development of neonicotinoid insecticides and applied for a lot of patents, but not many have become commercial products. This may be because the newly developed pesticides are still unable to match the price and activity when compared to imidacloprid and acetamiprid. Global sales, production and control of five major insecticides in 2014 are listed in Table 1.

## NIs Intermediates

2-Chloro-5-methylpyridine is a key intermediate for the synthesis of nicotinic insecticides such as imidacloprid and acetamiprid as well as a crucial intermediate for drugs and dyes. In terms of the synthetic route, chemical synthesis method possesses several drawbacks including complicated steps, by-products generation, serious pollution and high cost. Therefore, the synthesis method of 2-chloro-5-methylpyridine needs to be largely improved [15].

With the widespread utilization of nicotinic insecticides, the research on the synthesis of the intermediates of these insecticides also flourished, and the research on the hydroxylation of nicotinic acid has become a hot spot. Nicotinic acid can be directly converted into 6-hydroxynicotinic acid in one step by microorganisms, and then further chlorinated, followed by reduction to synthesize 2-chloro-5-chloromethylpyridine. This method is an effective combination of biotechnology and chemical technology, and provides a novel alternative for the synthesis of 2-chloro-5-chloromethylpyridine (Fig. 1). The research on the hydroxylation of nicotinic acid to generate 6-hydroxynicotinic acid brought about the production process improvement and technological progress of 2-chloro-5-chloropicoline [16]. Since microbial transformation route is occupied with the merits such as strong regioselectivity, high yield and environmental friendliness, it recently become a research central issue by scientists.

## 6-Hydroxynicotinic Acid

6-Hydroxynicotinic acid (6-HNA), also known as 6-hydroxy-pyridine-3-carboxylic acid, has alcoholic and keto structures. 6-HNA is acidic in aqueous solution and harbors low solubility, but it is easily soluble in alkaline solution [17]. At present, chemical methods were mainly employed for the industrial production of 6-HNA, usually involving in using of fumaric acid or malic acid as raw materials, and the reaction process requires concentrated sulfuric acid or concentrated hydrochloric acid and other reagents, which is easy to result in environmental pollution and the increase in production costs [18]. Therefore, the bioconversion of nicotinic acid to 6-HNA by nicotinate dehydrogenase possesses theoretical significance and practical value.

## Microbial Conversion of Nicotinate

### Nicotinate Dehydrogenase

Nicotinate dehydrogenase (NDHase, EC1.17.1.5), also known as nicotinic acid hydroxylase, is a kind of oxidoreductase widely existing in nature. Allinson et. al first found that some microorganisms were able to grow on medium with nicotinic acid as nutrients and isolated the intermediate 6-hydroxynicotinic acid [19]. Then, this reaction was confirmed as a dehydrogenation process, so the enzyme was defined as NDHase or nicotinic acid hydroxylase. Subsequent studies demonstrated that majority of NDHases can catalyze certain hydroxylation reactions toward some nitrogen-containing heterocyclic compounds of specific sites, which is the first step in the metabolism of nitrogen-containing heterocyclic compounds by lots of microorganisms, such as *Pseudomonas* spp., *Comamonas testosteroi* and so forth [20, 21]. NDHase exerts a crucial role in the metabolism of nitrogen-containing heterocyclic compounds and provides new directions as well as ideas for the preparation of some chemical intermediates which are difficult to synthesize.

## Sources

NDHase is mainly derived from several strains that could metabolize nitrogenous heterocyclic compounds. Currently, NDHase is basically from phylum *Proteobacteria*, including genus *Pseudomonas*, *Achromobacter*, *Serratia*, *Bacillus*, *Comamonas* and some anaerobic *Clostridium* which was summarized in Table 2. Among them, NDHase was the most densely distributed in genus *Pseudomonas* and *Comamonas*. Since its initial discovery and identification, NDHase has undergone a long exploration and research. It is worthwhile noting that Hunt *et al*. used H_2_O^18^ labeled with oxygen-18 as a solvent to catalyze the reaction based on the isotope tracer method in 1959, and discovered that the C6 of the product 6-HNA carried -^18^OH, which was labeled with oxygen-^18^O, confirming that the reaction catalyzed by NDHase was hydroxylation rather than mono-oxygenation reaction.

## Component and Catalytic Characteristics

Although NDHase is widely distributed, most of them possess multi-subunit structures and exist in the inner membrane of cells, which requires the electron transport chain to exert its function and activity [22, 23]. However, a few NDHase only have single subunit, which can also hydroxylate niacin. For example, Nagasawa *et al*. screened and obtained an NDHase with the size of 80 kD from *Pseudomonas* TN5 in 1994. After SDS-PAGE electrophoresis, the protein was observed to be a single band, and the enzyme was confirmed to be a membrane binding protein [24]. In contrast, lots of studies had been carried out on the composition of multi-subunit NDHase. Behrman *et al*. elucidated that NDHase was bound in cell membrane by separation and purification in 1957. It was located at the insoluble components after centrifugation, and further purified after re-dissolution by X-100, in which the results showed that the reaction of NDHase required the involvement of cytochrome in the electron transport system [25]. In 1959, NDHase from *Pseudomonas* was also purified and further confirmed to be a membrane binding protein as well as the insoluble component [26]. In addition, this study showed that the enzyme contains cofactors of cytochrome and flavoprotein. Nagel *et al*. purified NDHase from *Bacillus* sp. DSM2923 and observed that this enzyme was composed of three subunits with sizes of 20 kD, 34 kD and 85 kD, respectively [22]. Based on the absorption wavelength data, it was speculated that the subunit of 20 kD was the center of iron-sulfur protein, the subunit of 34 kD was the flavoprotein and the subunit of 85 kD was the molybdenum binding protein. Ashraf Alhaper *et al*. first reported the coding gene of NDHase purified from the strain *Eubcterium barkeri*, and discovered the gene cluster consisted of four genes, whose coding protein sizes were 23 kD, 33 kD, 37 kD and 50 kD respectively, corresponding to four subunits of *NdhS*, ndhF, *NdhM* and *NdhL*, respectively. Specifically, *NdhS* functions as an iron-sulfur protein domain [2Fe-2S], while ndhF serves as a flavoprotein. *NdhL* and *NdhM* correspond to two molybdenum binding proteins [27]. This result was consistent with the electron paramagnetic resonance analysis of purified NDHase by Dilworth *et al*. [28]. In recent years, Yang Yao *et al*. also reported a gene sequence of NDHase, which consisted of three gene segments forming a gene cluster, encoding three subunits of *NdhS*, *NdhL* and *NdhM*, respectively with sizes of 21 kD, 82 kD and 46 kD, and they function as iron-sulfur protein, molybdenum-binding protein and flavoprotein in turn [29]. Due to its extremely low expression level in wild strains, it is challenging to purify enough proteins for structural analysis. Furthermore, few studies currently have been reported on the crystal structure of NDHase because of its complex structure as well as membrane-bound protein [30].

Majority of NDHases can only react with C6 hydroxylation of nicotinic acid, such as *Serratia marcescens*, *Pseudomonas fluorescens* TN5, *Achromobacter xylosoxidans* and so forth, while a few of them harbor the capacity of both C6 hydroxylation of nicotinic acid and 3-cyanopyridine. The exploration of structure and function of NDHase is still in preliminary stage. These studies can provide theoretical guidance for further development and modification of NDHase.

## Function of Each Subunit

The hydroxylation of NDHase requires the participation of the electron respiratory chain system on the cell membrane, and the most important components of the electron respiratory chain are the hydrogen carrier, which is mainly composed of iron-sulfur proteins, NDHases, flavin dehydrogenase, ubiquinone (also known as coenzyme Q) and cytochromes. At present, the mechanism of hydroxylation reaction mediated by NDHase has not been effectively analyzed, but researchers have conducted in-depth studies on the functions of each subunit of NDHase in the electron respiratory chain.

**Fig. 2:**
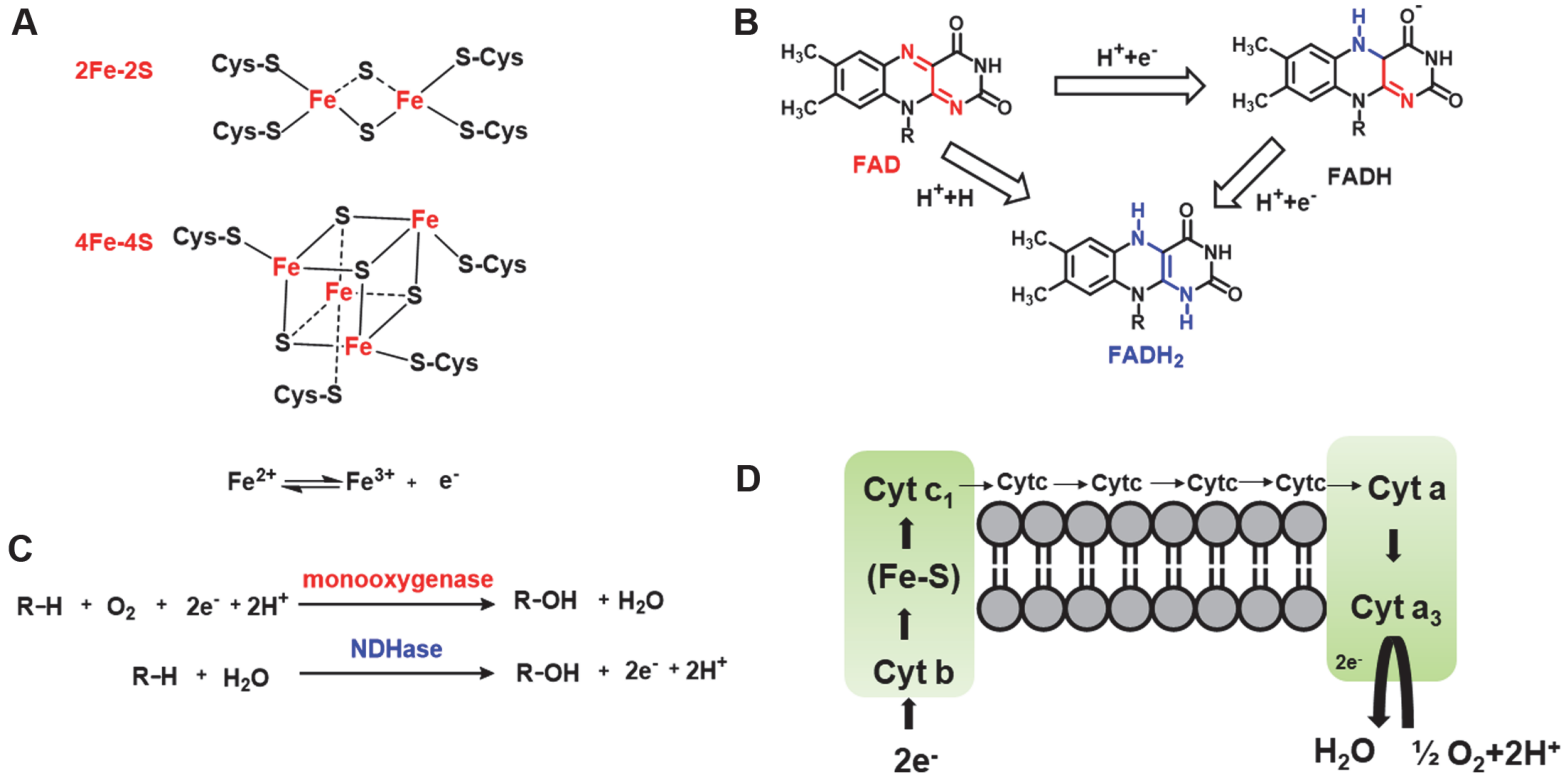
Main components of the electron respiratory chain is NDHase. (**A**) Schematic diagram of the structure of [Fe-S]. (**B**) Electron transfer mechanism of FAD. (**C**) Catalyzed reaction structural formula of monooxygenase and NDHase. (**D**) Electron transfer in cytochrome.

Large numbers of studies have suggested that most NDHases contain three subunits, namely iron-sulfur protein center [2Fe-2S], flavin protein and molybdenum binding protein [6]. Among them, iron sulfur protein center [2Fe-2S] and flavin proteins are hydrogen transmitters. The iron-sulfur protein contains acid-instable sulfur and non-porphyrin iron, which are arranged in a square or cube and form sulfur bridges with each other (Fig. 2A). It then forms a force with the cysteine of the protein to form an iron-sulfur protein. Since this active part contains an active iron as well as sulfur atoms, it is named the iron-sulfur center. Iron-sulfur proteins can form complexes with flavin proteins or cytochromes in mitochondria, and realize electron transfer by changing the redox state of iron in iron-sulfur center [31]. In the electron respiratory chain system, there are multiple iron sulfur centers involved in electron transfer.

Flavin protein contains flavin adenine dinucleotide (FAD) or flavin mononucleotide (FMN), both of which play a pivotal role in the electron respiratory chain of NDHase as protein cofactors [32]. Take FAD as an example (Fig. 2B), a pair of hydrogen removed from the substrate will combine with FAD to form FADH2, and nitrogen atoms at the first and tenth positions of the isoromazine ring on the structure of FAD can undergo repeated hydrogenation and dehydrogenation reactions to realize the electron transfer.

Molybdenum binding protein is another common structure in NDHase. Andreesen *et al*. believed that molybdenum binding protein could cleave water in solvent to produce e^-^ and H^+^ into the electron respiratory chain (Fig. 2C) [33]. Unlike the oxidation reaction of mono-oxygenase, the -OH produced by molybdenum binding protein after water splitting will nucleophilically attack the nitrogen atoms of nitrogen-containing heterocyclic compounds. Since the form of hydroxyl-pyridine at the 2,4,6 positions are easier to generate than the tautomeric pyridine form, NDHase generally hydroxylates at the 2,4,6 positions of nitrogen-containing heterocyclic compounds. The hydrogen and electrons produced will enter the iron-sulfur center, FAD or cytochrome in the electron respiratory chain for electron transport.

In addition, several NDHases use cytochrome as a prosthetic group for electron transport (Fig. 2D). Cytochromes are proteins that use heme or porphyrin iron as prosthetic groups. Although there are many kinds of cytochromes, only a few named b, cl, c, a and a3 currently be involved in the mitochondrial electron respiratory chain. Generally, the transfer order of electrons in the cytochrome is b-c1-c-a-a3-O_2_ in the respiratory chain. a and a3 cannot be separated now, so they are collectively called cytochrome oxidase. While most literature reports that NDHase contains more than one cytochrome c structure, which is involved in electron transport along with iron-sulfur centers [34]. To sum up, the general process of NDHase-catalyzed reaction can be divided into two parts. Firstly, the water is split by molybdenum binding protein to generate hydrogen and electrons. Secondly, the hydrogen and electrons enter the electron respiratory chain. FAD, cytochrome and iron-sulfur center of NDHase participate in the energy transfer.

## Heterologous Expression

At present, there are a few reports on the gene cloning and recombinant expression of NDHase. Ashraf Alhaper *et al*. first reported the NDHase gene from *Eubcterium barkeri* and investigated its structure and function, indicating that the coding gene of this enzyme is a gene cluster composed of four transcription-coupled genes on chromosomes [27]. The genes *NdhF*, *NdhS*, *NdhL* and *NdhM* encode four subunits, respectively, whose functions are flavin protein, iron-sulfur central domain and two molybdenum binding proteins, separately. However, they failed to conduct the study of heterologous recombinant expression. Yang *et al*. for the first time cloned the NDHase gene (Genbank accession: EU 604833) from *Pseudomonas putida* KT2440, which was then successfully expressed in *Pseudomonas entomophila* L48. This enzyme was able to complete the hydroxylation of C6 with nicotinic acid as the substrate, and subsequently confirmed that the subunit was the active center of the catalytic reaction through the knockout and recovery of the iron-sulfur center [29]. In addition to this, they also cloned the NDHase gene (Genbank accession: EU 604833) from *Comamonas testosteroni* JA1, and heterologous expression of this enzyme was achieved in *Pseudomonas entomophila* L48 [29]. It was observed that this enzyme could not only catalyze the hydroxylation of C6 in nicotinic acid, but also catalyze the hydroxylation of C6 in 3-cyanopyridine. On the one hand, only a few literatures presently reported the sequences of NDHase and majority of the them are produced by the wild strain, in which the protein structure and composition are preliminarily investigated by separation and purification. On the other hand, the enzymatic activity of wild strain is low when preparing the target products, and the expression of NDHases as well as the catalytic mechanism are not completely clear. Even if among a few NDHase sequences that have been reported, there are few highly efficient heterologous expression studies. Although NDHase has been cloned and expressed successfully, obvious obstacles exist in the expression of NDHases in *Escherichia coli*, a widely used protein heterologous expression model organism. All the expressions occurred in the form of inclusion bodies without hydroxylation activity. It is speculated that the expression process of this enzyme depends on some unusual protein expression mechanisms in wild strains, but lacks a similar expression mechanism in *E. coli*. At present, the successful functional expression was all carried out in *Pseudomonas* spp. As the host was not a model organism, the available genetic engineering tools and technical means were not mature and perfect, resulting in low expression activity of the recombinant enzyme.

## Advances in Catalytic Reactions to Pyridine Substrates

In recent years, it has been suggested that NDHase can catalyze a variety of pyridine substrates, such as 3-cyanopyridine, 3-pyridine sulfonate and nicotinic acid, to obtain C6 hydroxylation products, such as 3-cyano-6-hydroxy-pyridine, 6-hydroxy-3-sulfonate pyridine and 6-hydroxynicotinic acid. The substrate preference of NDHase from diverse sources also significantly varies. For instance, NDHase from *Pseudomonas fluorescens* TN5 and *Comamonas testosteroni* JA1 was both specific to 3-cyanopyridine and nicotinic acid [24, 35]. NDHase derived from *Comamonas teststeroni* JA1 is against hydroxylating pyridine 3-sulfonate. NDHase from *Pseudomonas putida* KT2440 and *Pseudomonas putida* NA-1 can only transfer nicotinic acid, but fail to 3-cyanopyridine [29, 36]. The hydroxylation product 3-cyano-6-hydroxypyridine catalyzed by NDHase toward 3-cyanopyridine is attractive since the cyano group can easily be converted to other groups by simple chemical methods, like ammonia methyl for secondary ammonia. In the production of pyridine heterocyclic nicotinoids such as imidacloprid, 3-cyanohydroxypyridine is more convenient for subsequent chemical reduction steps than 6-hydroxyniacin along with cheaper price than nicotinic acid. It is easy to biosynthesis of 6-hydroxynicotinic acid with the available NDHase while is limited for the low catalytic activity for 3-cyanopyridine. As is mentioned above in Table 1, several NDHases can only catalyze the hydroxylation of nicotinic acid, but not to 3-cyanopyridine. Although other NDHases were able to catalyze nicotinic acid and 3-cyanopyridine at the same time [29, 37], are more active toward nicotinic acid. For example, Yang *et al*. reported the production of 6-hydroxy nicotinic acid and 3-cyano-6-hydroxypyridine by double catalytic process coupling, with product concentrations reaching 50.38 g/l and 5.77 g/l, respectively [38]. The concentration of 3-cyano-6-hydroxypyridine exceeded 4.39 g/l produced by resting cells of *Comamonas testosteroni* MCI2848 according to the report by Yasuda *et al*. If nicotinic acid was not added during reaction, the concentration of 3-cyano-6-hydroxypyridine can only reach 2.25 g/l [39].

## Substrate Molecular Recognition Mechanism

Interactions between the advanced structure of NDHase and the substrate molecules, namely the distinguishment in the recognition ability of the substrates, determine the catalytic ability of NDHase for diverse substrates. The existing research focused mainly on the experimental results of the substrate specificity of NDHase. Only a superficial and empirical summary of its recognition with pyridine substrates is simply performed. It is speculated that the conjugate and inductive effects of the substituent group at position 3 of the pyridine ring exert a critical role in the hydroxylation reactions at position 6. The stronger the inductive and conjugate effects would result in an easier the reaction [38]. Nevertheless, few gene sequences of NDHase have currently been submitted, and the crystal structure of NDHase also has not been resolved so far. It is challenging to investigate the interaction between NDHase and various pyridine substrates at the molecular level based on the reliable spatial structure of NDHase. As a result, the molecular recognition mechanism of NDHase for pyridine substrates has not been effectively solved.

Therefore, studying and analyzing the recognition mechanism of NDHase toward pyridine substrates can provide theoretical guidance and technical support for the design, modification and construction of highly efficient NDHase to a certain extent. Based on the analysis of the molecular recognition mechanism, studies on the enhancement of selectivity of NDHase for the hydroxylation of 3-cyanopyridine through rational protein design should be carried out and a highly selective NDHase for the hydroxylation of 3-cyanopyridine would be constructed, significantly improving its catalytic activity for 3-cyanopyridine. This can not only deepen our understanding of the theory of catalytic mechanism, but also improve the catalytic activity of NDHase, which will greatly enhance the application value of NDHase.

## Application

6-HNA is a high-value pharmaceutical intermediate and fine chemical that is attracting widespread attention and has been successfully applied in the fields of pesticide and wastewater treatment.

### Pesticide Industry

NDHase is a kind of biocatalyst with important industrial application value. By using the hydroxylation reaction catalyzed by NDHase, the hydroxyl group can be introduced into the C6 position of the pyridine ring, and the pyridine chemical intermediates such as 6-hydroxyniacin, 3-cyano-6-hydroxypyridine can be obtained for the synthesis of important novel pyridine heterocyclic nicotinoid insecticides, such as imidacloprid, acetamiprid, thiacidacloprid and so forth. Due to its advantages of high efficiency, wide range of action and environmental friendliness, this new pyridine heterocyclic nicotinic agricultural insecticide has become a hot spot in the development of pesticides at present. It is gradually replacing the nicotinic pesticides with decreasing activity, and has become the main drug for killing aphids, planthopper and other pests, and the market capacity is rapidly expanding. Nicotine pesticides insecticides are a group of analogues derived from the nicotine from plants. Their main biological function is to act on acetylcholinesterase and damage the nerve conduction of harmful insects so as to kill insects. Traditional nicotine possesses lots of problems due to its low activity and great damage to the environment. Pyridine heterocyclic neonicotinoid insecticides are a new type of pesticide synthesized by structural modification of traditional nicotine to simulate plant-derived nicotine. It is a new milestone in the development history of insecticides after organophosphorus, pyrethroid, carbamate and other insecticides, and has become one of the most important insecticide varieties. However, the chemical synthesis of these pyridine heterocyclic intermediates has complicated steps, lots of side reactions and serious pollution. The preparation of these pyridine derivatives catalyzed by NDHase harbors the advantages of high specificity, mild conditions and high yield. Therefore, it has low cost, high efficiency and green process, which has attracted wide attention. Take the most widely used imidacloprid as an example, 2-chloro-5-chloromethylpyridine is a crucial intermediate in the synthesis of imidacloprid. There are relatively several methods for the chemical synthesis of 2-chloro-5-chloromethylpyridine, such as the synthesis of 2-chloro-5-chloromethylpyridine from 3-methylpyridine and dehalogenation reaction is then conducted to form 2-chlor-5-chloromethylpyridine [40]. But this method has low yield, high catalyst cost and serious pollution. The synthesis of 2-amino-3-methylpyridine from 3-methylpyridine was relatively good, then the amino group was replaced by-Cl, and then 2-chloro-5-chloromethylpyridine was synthesized. Although several previous steps of this reaction have a relatively high yield, the side reactions are more and the product separation is more difficult. Another method is concerned with using 3-methylpyridine to synthesize nicotinic acid, with subsequent hydroxylation reaction of nicotinic acid C6, and finally replace the carboxyl group to produce 2-chloro-5-chloromethylpyridine. This method also has many side reactions, such as difficult product separation, and low yield.

The one-step catalytic hydroxylation of C6 position of nicotinic acid or 3-cyanopyridine can be achieved by microbial method (Fig. 1), and almost no by-products are generated, so it is easy to separate and purify. The C6 hydroxyl group of 6-hydroxy nicotinic acid or 6-hydroxy-3-cyanopyridine is easily replaced by Cl atoms, and the carboxyl group or cyano group at C3 is easily replaced by chloromethyl to form 2-chloro-5-chloropyridine. Mitsubishi, for example, has successfully commercialized this biocatalytic process. 3-Cyano-6-hydropyridine was prepared and used in the production of imidacloprid.

### Wastewater Treatment

At present, there are many nitrogen-containing heterocyclic compounds in the environment, especially since most of the aromatic compounds produced and discharged in industry are nitrogen-containing heterocyclic compounds. These compounds are widely used in textile industry, pharmaceutical API production, pesticide and other industries. Quinoline and pyridine, including their derivatives, are representative nitrogen-containing heterocyclic compounds, widely present in the wastewater of these industries, with strong toxicity and irritating odor, its carcinogenicity and teratogenicity cannot be underestimated. Due to the great toxicity, the degradation of these compounds is greatly urgent. The use of microorganisms to treat sewage and pollutants is a research hotspot nowadays. Using existing treatment facilities, adding NDHase producing strains to wastewater treatment systems can improve treatment capacity. For example, Padoley *et al*. listed several common nitrogen-containing heterocyclic compounds in sewage and the strain *Pseudomonas putida* QP2 was used for biological wastewater treatment, good results were achieved which indicated that 6-methylquinoline was degraded at 650 mg/l concentration within 24 h at 28°C [41]. Johansen *et al*. reported the utilization of strain *Desulfobacterium indolicum* to metabolize quinoline and isoquinoline to purify sewage [42].

### Outlook and Future Perspectives

So far, although there have been several reports on the production of NDHase strains as well as few of them was successfully applied to the production of novel nicotine pesticide intermediates, lots of issues still existed in the production of screened wild strain. In addition, the NDHase is kind of membrane-protein and the enzyme system is extremely complex. Nevertheless, generated by-products by NDHase would affect the subsequent separation and purification, reducing the yield of the reaction and increasing post-processing costs. On the other hand, due to the unclear genetic background of wild strains, the low expression level of enzymes, and the lack of corresponding genetic manipulation tools, it is more blind and difficult to modify them. To sum up, as far as I am concerned, future work about NDHase could focus on the following points: (I) the number of screened hosts and expression plasmids is limited, and it is necessary to continue to screen suitable expression systems. (II) the overall expression of NDHase is not high, and the enzyme activity is quite low, so the research can continue to improve the expression of each subunit. (III) due to the complicated structure of NDHase, it is a pivotal direction for further study to analyze its crystal structure and catalytic mechanism by using cryo-electron microscopy. (IV) the catalytic efficiency and stability of wild and recombinant types of NDHase need to be substantially improved, and efficient immobilization investigation can be considered.

## Figures and Tables

**Table 1:** Global sales, production and control of five major insecticides in 2020.

Types	Sales in 2020 (US dollars billion)	CAGR (%,2015-2020)*	Manufacturer	Applicable crops	Object of control
Thiamethoxam	1.18	9.6	Syngenta	Soybeans, fruits, corn, vegetables, grains, cotton	Diverse aphids, leafhoppers, beetles
Imidacloprid	1.16	4.1	Bayer	Soybeans, fruits, vegetables,	Diverse aphids, rice planthoppers
Clothianidin	0.46	7.2	Bayer	Sumitomo Corn, canola, grains	Diverse aphids, planthoppers
Acetamiprid	0.27	7.9	SODA	Corn, vegetables, rice	Diverse aphids, needleworm, red spider
Thiacloprid	0.15	7.4	Bayer	Canola, Corn, vegetables	Diverse aphids, rice planthoppers, Thrips

*Note: CAGR represents compound annual growth rate.

**Table 2:** Summaries of reported NDHase producing strains.

Strains	Optimum temperature (°C)	Optimum pH	Optimum substrate concerntion (%)	Substrate specificity	Enzyme activity (U/ml)	Reference
*Bacillus* sp. DSM 2923	-	-	-	NA	2.2 U/mg	[22]
*Clostridium barkeri*	-	-	-	NA	-	[43]
*Comamonas testosteroni JAl*	30	7.0	3.0	3-CP, NA	0.42	[35]
*Eubacterium barkeri*	-	-	-	NA	-	[27]
*Pseudomonas putida* S14	50	5.5	3.0	3-CP, NA	1.11	[44]
*Pseudomonas putida* H9	25	7.0	1.0	3-CP, NA	0.37	[45]
*Pseudomonas putida* NA-1	30	7.0	3.0	NA	0.58	[36]
*Pseudomonas putida* BKC4	30	7.0	1.0	NA	0.53	[46]
*Pseudomonas putida* KT2440	30	7.0	2.0	NA	0.34	[29]
*Pseudomonas fluorescens* KB1	30	7.2	-	NA	-	[26]
*Pseudomonas fluorescens* TN5	28	7.0	1.0	NA	5.33 U/mg	[24]
*Pseudomonas* sp. BK-1	30	7.0	1.25	NA	0.57	[47]
*P. entomophila* L48	30	7.0	2.0	NA	0.033	[29]
*Serratia marcescens IFO12648*	28	7.0	1.0	NA	-	[37]

“-” represents experiments that were not conducted by authors. 3-CP represents 3-cyanopyridine. NA represents nicotinic acid.
